# Differential Expression of Six *Rnase2* and Three *Rnase3* Paralogs Identified in Blunt Snout Bream in Response to *Aeromonas hydrophila* Infection

**DOI:** 10.3390/genes9020095

**Published:** 2018-02-14

**Authors:** Ruijing Geng, Han Liu, Weimin Wang

**Affiliations:** 1 Key Lab of Agricultural Animal Genetics, Breeding and Reproduction of Ministry of Education/Key Lab of Freshwater Animal Breeding, College of Fisheries, Huazhong Agricultural University, Wuhan 430070, China; gengruijing@webmail.hzau.edu.cn; 2 Key Laboratory of Freshwater Fisheries and Germplasm Resources Utilization, Chinese Academy of Fishery Sciences, Wuxi 214081, China; 3 Collaborative Innovation Center for Efficient and Health Production of Fisheries in Hunan Province, Changde 415000, China

**Keywords:** blunt snout bream, *Rnase2*, *Rnase3*, expression patterns, biological function

## Abstract

Ribonucleases (*Rnases*)*2* and *Rnase3* belong to the ribonuclease A (RnaseA) superfamily. Apart from their role in molecular evolutionary and functional biological studies, these genes have also been studied in the context of defense against pathogen infection in mammals. However, expression patterns, structures and response to bacterial infection of the two genes in blunt snout bream (*Megalobrama amblycephala*) remain unknown. In this study, we identified multiple copies of *Rnase2* (six) and *Rnase3* (three) in the *M. amblycephala* genome. The nine genes all possess characteristics typical of the RnaseA superfamily. No expression was detected in the early developmental stages, while a weak expression was observed at 120 and 140 h post-fertilization (hpf) for *Rnase2b*, *Rnase2c*, *Rnase2e* and *Rnase3a*, suggesting that only three copies of *Rnase2* and one of *Rnase3* are expressed. Interestingly, only *Rnase2e* was up-regulated in the kidney of *M. amblycephala* after *Aeromonas hydrophila* infection, while *Rnase3a* was significantly up-regulated in liver, gut and blood after the infection. We conclude that the paralogs of *Rnase3* are more susceptible to *A. hydrophila* infection than *Rnase2*. These results indicate that different *Rnase2* and *Rnase3* paralogs suggest a role in the innate immune response of *M. amblycephala* to bacterial infection.

## 1. Introduction

The ribonuclease A (RnaseA) superfamily, also known as the pancreatic ribonuclease (Rnase) family, is the sole vertebrate-specific enzyme family [1,2]. The superfamily has been studied rather extensively in mammals, including humans, cattle, monkeys and mice, but studies in fishes remain comparatively scarce. In fishes, Rnase1 to 5 from *Danio rerio* [3,4,5,6], Rnase1 to 3 from *Oryzias latipes* and Rnase1-2 from *Salmo salar* [7] have been reported. All members of this superfamily have a few common characteristics: the entire coding sequence is contained within a single exon, a peptide signal at the N-terminal that directs protein biosynthesis within the endoplasmic reticulum, 6–8 conserved cysteines which form disulfide bonds, a catalytic triad composed of the conserved “CKXXNTF” functional motif, and finally two histidine residues and one lysine residue with catalytic activity [8]. However, physiological functions of the superfamily members, as well as their expression patterns in tissues, are distinct. *Rnase1* is expressed in the pancreas, brain and kidney; *Rnase4* mainly in liver and gut; *Rnase5* in liver and testis; and *Rnase6* in liver and brain [9]. RnaseA superfamily members have a wide variety of different functions, including the degrading of dietary RNAs [1], antibacterial activities [10], stimulation of blood vessel formation [11], host defense [12], and reproductive functions [13,14].

Rnase2 is a cytotoxic protein secreted together with the Rnase3 by cytoplasmic granules of eosinophils in mammals [15]. It is known to be expressed in the liver, spleen, placenta in humans and ruminants [1,16]. In mammals, Rnase2 shows the specific antiviral activity *in vitro* experiments [17]. Rnase2 was able to reduce the infection rate of the respiratory syncytial virus (RSV) and human immunodeficiency virus (HIV) in cell culture experiments [18,19]. Rnase2 exhibits chemotactic properties and plays an important role in the regulation of autoimmunity [20]. However, the expression patterns and biological functions of Rnase2 in mammals are different from the fish species. In fishes, gene expression analysis showed that *D. rerio Rnase1* and *Rnase2* are mainly in the adult liver and gut, and weakly expressed in the heart. Functional analysis of recombinant proteins demonstrated that *D. rerio* Rnases have strong antibacterial activity *in vitro* [3]. *Rnase1* and *Rnase2* in adult *S. salar*, mainly expressed in the thymus, have both angiogenic and bactericidal activities [7]. Rnase2 has 100-fold higher ribonuclease activity than Rnase3 [21]. 

Rnase3 is a host defense ribonuclease involved in inflammatory responses in humans. Molecular cloning experiments confirmed that both Rnase2 and Rnase3 (67% amino acid sequence similarity) are members of the RnaseA superfamily [22], and both have since been shown to be active ribonucleases [23]. Rnase3 exhibits high antibacterial activity against both Gram-positive and Gram-negative bacteria, as well as antiviral activity and antiparasitic functions against helminths and protozoa [24,25,26]. In fishes, *Rnase3* of *D. rerio* is highly expressed in heart and gut, and has bactericidal activity [3]. *Rnase1* gene, a member of RnaseA superfamily, has multiple copies. Duplications of *Rnase1* have been observed in *Myotis altarium* (*Rnase1α*, *Rnase1β* and *Rnase1γ*), *Martes flavigula* (*Rnase1-1*, *Rnase1-2* and *Rnase1-3*), *Callosciurus prevostii* (*Rnase1α* and *Rnase1β*), and *Colobus guereza* (*Rnase1α*, *Rnase1β* and *Rnase1γ*), etc. [27,28,29]. However, there is no genetic duplication of *Rnase1*, *Rnase2 and Rnase3* in teleost. It has been suggested that these varied functions emerged after gene duplication under a positive Darwinian selection [27].

The blunt snout bream (*Megalobrama amblycephala*) has recently become one of the economically most important freshwater cultured fishes in China. It is recognized as an ecofriendly and resource-conserving fish because of its fast growth, herbivorous diet, ease and low costs of breeding and high nutritional value. With rapid development of the *M. amblycephala* aquaculture industry, infectious diseases are becoming increasingly prevalent, resulting in substantial economic losses [30,31]. *Ma*-*Rnase1* has been cloned and characterized, and its recombinant protein was found to have digestive activity and antibacterial function [29]. Recently, our laboratory has published the entire genome of this species [32], so now we can identify all members of the RnaseA superfamily present in its genome. Here, we identified six *Rnase2* paralogs (*Rnase2a*, *Rnase2b*, *Rnase2c*, *Rnase2d*, *Rnase2e* and *Rnase2f*) and three *Rnase3* paralogs (*Rnase3a*, *Rnase3b* and *Rnase3c*) in the genome of *M. amblycephala*. Three-dimensional structures of all proteins were modeled and compared. The messenger RNA (mRNA) expression levels of all nine genes (6+3) were determined by quantitative polymerase chain reaction (qPCR) in the early developmental stages, as well as in different tissues of one-year-old and two-year-old *M. amblycephala*. In order to infer their functional roles, we also studied the expression patterns of these genes in five different tissues at 6 and 12 h after an immune-challenge with *Aeromonas hydrophila*. Our results are not only useful for the research of Rnase2 and Rnase3 evolution, but also help further elucidation of the regulation mechanisms in the innate immune response of *M. amblycephala* to bacterial infection.

## 2. Materials and Methods

### 2.1. Collection of Samples

Sample collection and experiments were conducted in accordance with the national legislation of China and approved by the ethics committee of Huazhong Agricultural University (No. HZAUFI-2017-006).

Healthy one-year-old (*n* = 10) and two-year-old (*n* = 70) *M. amblycephala* specimens, as well as three female and three male adult specimens for obtaining embryos via artificial breeding, were obtained from the Tuanfeng fish farm (Huanggang city, Hubei province, China), and kept in tanks at the Freshwater Fish Genetics Breeding Center of Huazhong Agricultural University (Wuhan, China). The fish (one- and two-year-old) were acclimated for two weeks, while maintaining water temperature at 26 ± 2 °C and an abundant oxygen level. They were fed commercial pellet feed twice daily for seven days before the onset of the study. Healthy two-year-old fish were divided into three groups: 20 “blank” (non-injected), 30 “challenge” (*A. hydrophila* injected) and 30 “control” (phosphate-buffered saline (PBS) injected). After the specimens from the blank group were anesthetized with 100 mg/L tricaine methane sulfonate (MS-222) and sterilized with 75% alcohol, eight type-tissue samples were immediately collected under sterile conditions from one- and two-year-old (adult) *M. amblycephala*: brain, heart, liver, spleen, kidney, muscle, gut, and testis. Seven different early developmental embryonic stages were also sampled: fertilized egg (0 h post-fertilization (hpf)), late gastrula stage (16 hpf), heart appearance (38 hpf), hatching (48 hpf), eye appearance (88 hpf), air bladder formation (120 hpf) and intestine appearance stage (140 hpf). All samples collected were immediately frozen in liquid nitrogen and stored at −80 °C.

### 2.2. Identification of Rnase2 and Rnase3 Genes

The coding sequences of *Ma-Rnase1*, *Ma-Rnase2* and *Ma-Rnase3* were obtained from our previous transcriptomic data for *M. amblycephala* (GenBank accession numbers: SRX731259 and SRA045792). The full-length sequences of *Ma-Rnase1*, *Ma-Rnase2* and *Ma-Rnase3* were obtained from the genome data of *M. amblycephala* (Figure S1).

### 2.3. Protein Alignment and Physicochemical Properties

The protein sequences of *Ma-Rnase1*, *Ma-Rnase2* and *Ma-Rnase3* were inferred from the genome data of *M. amblycephala*. The protein sequences of *Danio rerio* used for the analysis were obtained from the GenBank: *Rnase1* (ABQ23783.1), *Rnase2* (ABQ23784.1) and *Rnase3* (ABQ2385.1). Orthologs were then aligned by CLC Sequence Viewer 6.8 (Qiagen, Hilden, Germany). Physicochemical properties, as well as functional sites and domains, of all nine proteins (Rnase2a, Rnase2b, Rnase2c, Rnase2d, Rnase2e, Rnase2f, Rnase3a, Rnase3b and Rnase3c) were predicted using different tools available from the ExPASy web server [33]. SignalP 4.1 server [34] was used to predict the signal peptide cleavage sites of the N-terminals of proteins. The isoelectric point (pI) and molecular weight (Mw) of the mature proteins were calculated with the ExPASy online tool Compute pI/Mw.

### 2.4. Structural Predictions

Three-dimensional structures of proteins were modeled with the SWISS-MODEL server, selecting nine gene models in the protein structure model database to build the models [35]. The sequence identity values between the template (*Rnase1*) and the nine studied genes (*Rnase2a-f* and *Rnase3a-c*) were 60%, 62%, 58%, 50%, 57%, 50%, 33%, 33% and 25%, respectively. Next, the secondary structures of *Ma*-Rnase1-3 protein sequences were created using ESPript software [36]. Superimposed three-dimensional structures and functional sites of the nine studied genes (with template) were modeled by PYMOL (DeLano Scientific) [37]. 

### 2.5. Phylogenetic Analysis

MEGA 7.0.21 [38] was used to conduct phylogenetic analysis aimed to better understand the evolutionary relationships of Rnase1-3 among the teleosts (*M. amblycephala*, *D. rerio*, *O. latipes*, *S. salar*, *Oncorhynchus mykiss*) and mammals *(Bos taurus*, *Mus musculus*, *Homo sapiens*, *Rattus norvegicus*, *Macaca mulatta* and *Colobus angolensis*). All species sequences were downloaded from the GenBank [39]. Amino acid sequences were aligned with the Muscle program. The phylogenetic tree was reconstructed using the Neighbor-Joining (NJ) method with the protein Jones-Taylor-Thornton (JTT) matrix model and 1000 bootstrap replications [40].

### 2.6. Quantitative Analysis of Rnase2 and Rnase3 Messenger RNA Expression

Total RNA was isolated from each sample using TRIzol Reagent (TaKaRa Bio Inc., Dalian, China), following the manufacturer’s instructions. All RNA samples were treated with RNase-free DNase (TaKaRa Bio Inc.). The quality and quantity of RNA were evaluated with 1% agarose gel electrophoresis and the NanoDrop ND-2000 spectrophotometer (Thermo Fisher Scientific, Waltham, MA, USA), and then stored at −80 °C. We amplified the mRNA sequences of these nine genes with the primers presented in Table 1. The primers for the reference gene *β-actin* were 5’-ACCCACACCGTGCCCATCTA-3’ (forward) and 5’-CGGACAATTTCTCTTTCGGCTG-3’ (reverse) [41]. The first-strand cDNA was synthesized using the Prime Script™ RT reagent Kit with genomic DNA (gDNA) eraser (TaKaRa Bio Inc.), according to the manufacturer’s instructions. To analyze the expression patterns of *Rnase2* and *Rnase3* in healthy fish tissues and developing fish, all samples were analyzed using Light Cycler^®^ 480 II qPCR detection system (Roche Diagnostics, Mannheim, Germany). qPCR analysis were performed in a 96-well plate, where each well contained 20 μL of reaction mixture consisting of: 10 μL SYBR^®^ PreMix Ex Tapm II (TaKaRa Bio Inc., Dalian, China), 0.4 μL each primer (10 μM), 2 μL complementary DNA (cDNA) template and 7.2 μL sterilized double distilled water (ddH_2_O). The qPCR conditions were as follows: pre-denaturation at 95 °C for 5 min, followed by 40 cycles of amplification at 95 °C for 10 s, 60 °C for 10 s, and 72 °C for 15 s. Each sample was tested in triplicate. The average value per gene was calculated from three replicates. Finally, the expression of each gene was calculated using the 2^−^^△△Ct^ method [42].

### 2.7. Bacterial Challenge Experiment

For the bacterial challenge experiment, *A. hydrophila* was obtained from the Laboratory of Aquatic Medicine of the College of Fisheries at Huazhong Agricultural University (Hubei, China). *A. hydrophila* was incubated at 28 °C for 24 h on a Luria-Bertani Medium (LB) plate. We intraperitoneally injected the experimental group (two-year-old healthy *M. amblycephala*) with 1.0 × 10^7^ colony-forming units/mL of *A. hydrophila* (0.1 mL). The control group (two-year-old healthy *M. amblycephala*) was injected with 0.1 mL of sterilized PBS (pH 7.4). Six and twelve hours post-injection (hpi), each group was anesthetized with 100 mg/L MS-222 and dissected. Liver, spleen, kidney, gut and blood samples were then collected from each fish at 6 and 12 h after injection and control group. All samples collected were immediately frozen in liquid nitrogen and stored at −80 °C for RNA extraction and subsequent qPCR.

### 2.8. Statistical Analyses

All quantitative data are presented as the means of three individual experiments ± standard errors (SE). Statistical analyses were performed using SPSS 19.0 software (IBM Analytics, Richmond, VA, USA). Significant differences among samples were determined by the one-way analysis of variance (ANOVA) using least significant difference (LSD)'s multiple range test. *p* < 0.05 and *p* < 0.01 were considered as statistically significantly and highly significant, respectively. After analyzing the data, Origin9.0 mapping software (OriginLab, Northampton, MA, USA) was used to produce the expression map.

## 3. Results

### 3.1. Protein Alignment

**Figure 1:**
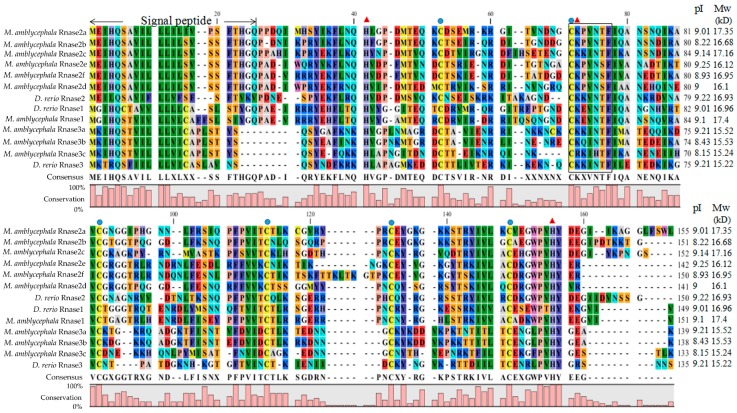
Multiple protein sequence alignments of Rnase1-3 in *Megalobrama amblycephala* and *Danio rerio*. Dashes indicate alignment gaps. The locations of the signal peptides are marked between the first two arrows. The isoelectric points and molecular weights (kDa) of the mature peptides are indicated by pI and Mw, respectively. The conserved “CKXXNTF” motif is boxed. The positions of six structural cysteines (active-site residues) are marked with blue circles. The three red triangles show the three catalytic residues.

The alignment of *M. amblycephala* and *D. rerio* Rnase superfamily members revealed that all proteins possess a structure common to the RnaseA superfamily, including a specific signal peptide of more than 20 amino acids at the N-terminal, coding sequences encoded on a single exon, the conserved “CKXXNTF” signature motif, two histidine residues and a lysine residue constituting a conserved catalytic triad, and six cysteines forming disulfide bonds (Figure 1). The calculated putative pI and Mw of the mature peptides of all nine genes in *M. amblycephala* and *D. rerio* were ranging from 8.15 to 9.25 and 15.22 to 17.39 kD, respectively (Figure 1). Moreover, we calculated the number of lysines and arginines in Rnase1 to 3 of fishes, humans and cattle (Table S1).

### 3.2. Structural Predictions

**Figure 2:**
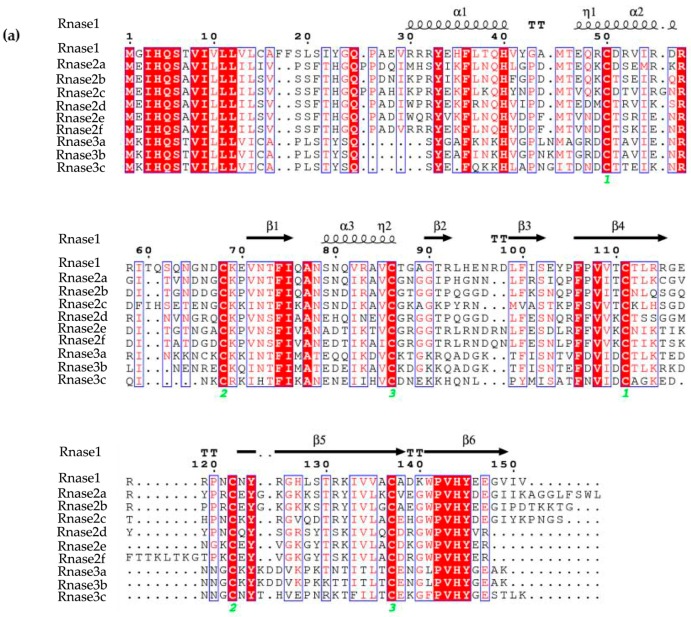

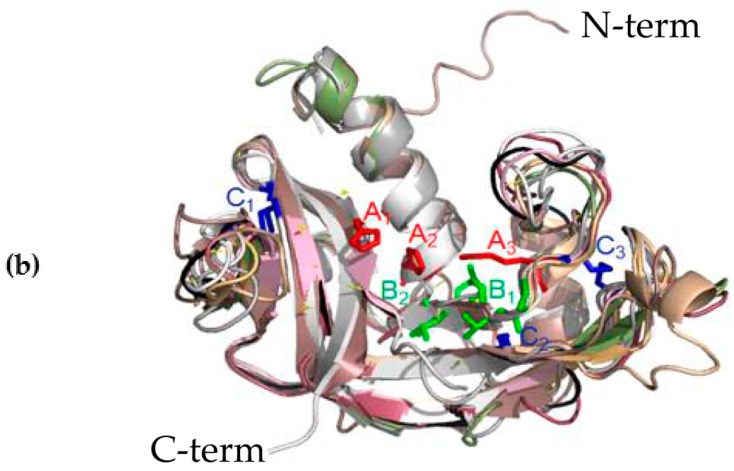
(**a**) Sequences alignment of *M. amblycephala Rnase1*, *Rnase2a*, *Rnase2b*, *Rnase2c*, *Rnase2d*, *Rnase2e*, *Rnase2f*, *Rnase3a*, *Rnase3b* and *Rnase3c*. Secondary structure elements of Rnases are depicted at the top. Cysteine pairings (disulfide bridges) are numbered in green below the columns. Locations of three α-helices (α1-α3) and six β-strands (β1-β6) are shown above the sequences. The figure was created using ESPript software. (**b**) Representation of the superimposed three-dimensional structures of all ten Rnases of *M. amblycephala*. The image was drawn using PYMOL. Catalytic residue regions are shown in red (A1-A3), conserved binding sites in green (B1, B2), and disulfide bond binding sites in blue (C1-C3).

The three-dimensional (3D) structures of *M. amblycephala* Rnase1-3 proteins were predicted by homology modeling. Their alignment shows a typical fold with three α-helices and six β-strands for members of the RnaseA superfamily (Figure 2A). Moreover, all studied proteins show a high level of conservation and high homology. The high conservation is observed in the conserved positions of three catalytic residues (A1, A2 and A3 in Figure 2B), and pyrimidine (B1) and purine (B2) binding sites. More variability was seen in disulfide bond binding sites (C_1_, C_2_, C_3_). Three-dimensional structures of individual proteins are available from the Figure S2.

### 3.3. Phylogenetic Analysis

**Figure 3:**
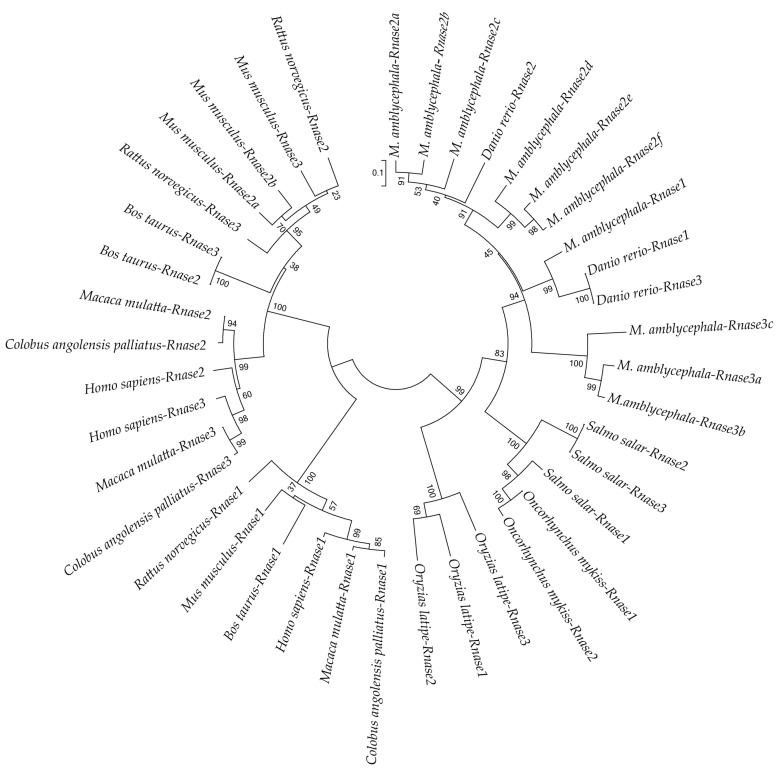
Neighbor-Joining (NJ) phylogenetic tree showing the relationships among protein sequences of *Rnase1*, 2 and 3 of various bony fishes and mammals. Numbers at the branches indicate bootstrap probabilities inferred with 1000 replicates.

To better understand the evolutionary relationships of Rnase1 to 3 in vertebrates, we reconstructed a protein sequence-based NJ phylogenetic tree of homologs in Teleosts and mammals. The two groups (fish and mammals) are divided into two distinct clades (Figure 3). The mammalian clade is further clearly divided into Rnase1 and Rnase2+Rnase3 branches. In the fish clade, *Ma-*Rnase1 and *Ma-*Rnase2 protein sequences are more similar to the corresponding orthologs belonging to *D. rerio* than to those of *O. mykiss*, *O. latipes* or *S. salar*. Rnase2 and Rnase3 of many species clustered together, including *M. amblycephala*, *S. salar*, *B. taurus* and *M. musculus*, which is a consequence of gene duplication. *M. amblycephala* has multiple copies of *Rnase2* and *Rnase3*, both of which formed their respective clusters. Because the sequences of Rnase1 to 3 are short and quite divergent, the bootstrap values on the phylogenetic tree are not high at some nodes.

### 3.4. Rnase2 and Rnase3 Messenger RNA Expression Patterns

**Figure 4:**
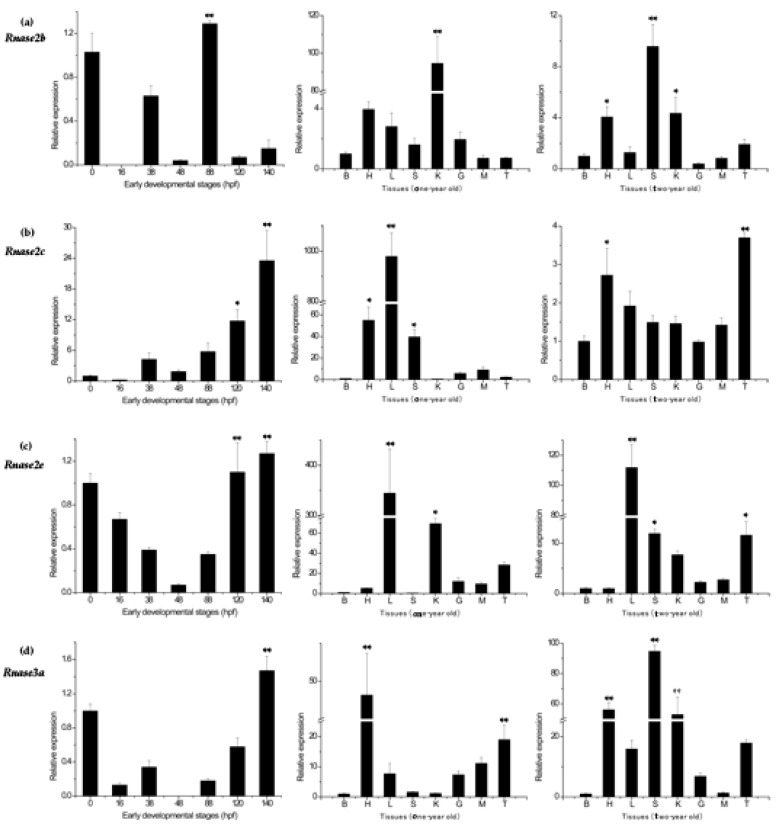
Expression patterns of (**a**) *Rnase2b*, (**b**) *Rnase2c*, (**c**) *Rnase2e* and (**d**) *Rnase3a* genes in different developmental stages and tissues of *M. amblycephala*. *β-actin* was used as the reference gene. Tissues: brain (B), heart (H), liver (L), spleen (S), kidney (K), gut (G), muscle (M), and testis (T). Developmental stages: 0 h post-fertilization (hpf) (fertilized egg), 16 hpf (late gastrulastage), 38 hpf (heart appearance), 48 hpf (hatching), 88 hpf (eye appearance), 120 hpf (air bladder formation) and 140 hpf (intestine appearance). The expressions in different developmental stages were normalized to the fertilized egg stage, while expressions in tissues were normalized to the expressions in brain tissue (both set as 1, or control group). Differences were statistically analyzed using one-way analysis of variance (one-way analysis of variance (ANOVA)). Highly significant differences from the control group (*p* < 0.01) are marked with **, and significant differences (*p* < 0.05) with *.

Expression of only four genes (*Rnase2b*, *Rnase2c*, *Rnase2e* and *Rnase3a*) could be detected in the eight different tissues of healthy *M. amblycephala* and during the early developmental stages (0, 16, 38, 48, 88, 120 and 140 hpf). No expression was detected for *Rnase2a*, *Rnase2d*, *Rnase2f*, *Rnase3b* or *Rnase3c*. The expression levels in the fertilized egg stage (0 h) were used as the standard against which the relative expression at other stages of development was calculated. *Ma-Rnase2b* was strongly expressed at 88 hpf, but weakly expressed at 120 and 140 hpf, with no expression at all at 16 hpf. However, *Ma-Rnase2c* and *Ma-Rnase2e* were strongly expressed at 120 and 140 hpf. During the remaining stages, expression levels were lower than during the fertilized egg stage (Figure 4). As the lowest expression was detected in brain tissue, expression levels in other tissues were normalized to this tissue. In the studied tissues of one-year-old fish, the highest expression levels of *Ma-Rnase2b* were detected in kidney (>90×), significantly higher (*p* < 0.01) than in any other tissue. *Ma-Rnase2c* and *Ma-Rnase2e* were expressed most highly in liver (979×, 344×, respectively) and *Ma-Rnase3a* was strongly expressed in heart (48×) and testis (19×). However, the expression patterns of the four genes in the two-year-old fish were dramatically different from those in the tissues of one-year-old fish. In the studied tissues of two-year-old fish, significantly higher (*p* < 0.01) expression levels of *Ma-Rnase2b* were detected in spleen (>9×). *Ma-Rnase2c* was expressed most highly in testis (>3.5×), followed by heart (>2.5×) and liver (>2.5×). *Ma-Rnase2e* was expressed in liver (>110×), followed by spleen (>10×) and testis (10×). *Ma-Rnase3a* was significantly higher (*p* < 0.01) expressed in spleen (>90×), heart (>55×) and kidney (>50×). The *Rnase3* showed an expression pattern different from *Rnase2*. The highest expression levels of four genes were detected in heart, spleen, testis, liver and kidney tissues. Detailed cycle threshold (Ct) values can be found in Table S2.

### 3.5. Expression Profiles of Rnase2 and Rnase3 Messenger RNA after Aeromonas hydrophila Challenge

**Figure 5:**
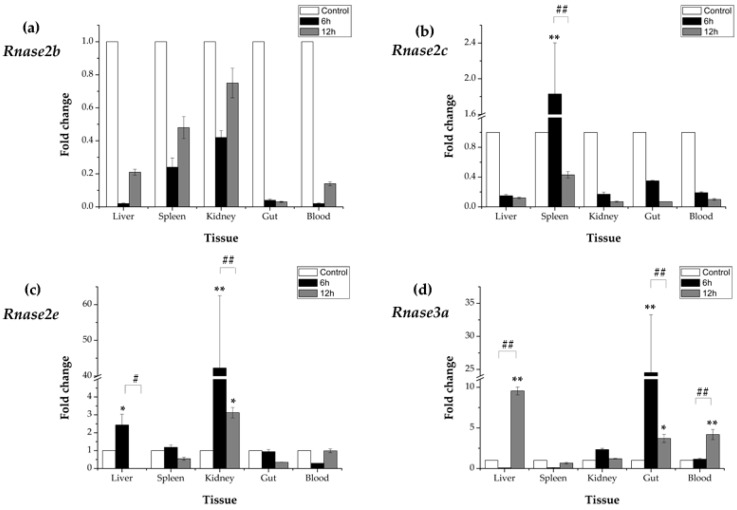
Messenger RNA (mRNA) after *Aeromonas hydrophila* infection in five different tissues (**a**) Expression of *Rnase2b*, (**b**) *Rnase2c*, (**c**) *Rnase2e* and (**d**) *Rnase3a*. *β-actin* was used as the internal reference. Results are presented as the mean ± standard error among three replicates, where significant differences (*p* < 0.05) between control and test groups are marked with *, and highly significant differences (*p* < 0.01) with **. # *p <* 0.05 and ## *p <* 0.01 show significant differences between the designated groups. 6h: 6 h after *A. hydrophila* infection; 12h: 12 h after *A. hydrophila* infection.

After an *A. hydrophila* immune-challenge, time-dependent relative (to the control group) expression patterns of *Rnase2* and *Rnase3* mRNA were observed in all five studied tissues (liver, spleen, kidney, gut and blood). The expression of *Rnase2b* showed down-regulated at 6 hpi (in comparison to the control) in liver (0.02×), spleen (0.24×), kidney (0.42×), gut (0.04×) and blood (0.02×) samples, and down-regulated at 12 hpi in liver (0.21×), spleen (0.48×), kidney (0.75×), gut (0.03×) and blood (0.14×) samples (Figure 5a), while the differences were not significant (*p* > 0.05). The expression of *Rnase2c* was only significantly (*p* < 0.01) up-regulated (1.83×) in spleen at 6 hpi compared to control, while down-regulated at 12hpi compared to 6hpi (*p* < 0.01). No significant differences (*p* > 0.05) were found in other tissues (Figure 5b). The expression of *Rnase2e* was significantly (*p* < 0.01) increased in kidney at 6hpi (42.27×) and 12 hpi (3.12×), followed by liver at 6hpi (2.44×) (*p* < 0.05), while no differences were found in spleen, gut and blood (*p* > 0.05) (Figure 5c). The expression of *Rnase3a* showed up-regulated at 6hpi in kidney (2.33×) and blood (1.16×), significantly up-regulated (*p* < 0.01) in gut (24.53×), but down-regulated in liver (0.07×) and spleen (0.09×) (*p* > 0.05) compared to control. However, at 12 hpi, the expression was significantly increased in liver (9.54×) (*p* < 0.01), blood (4.18×) (*p* < 0.01) and gut (3.71×) (*p* < 0.05) in comparison to control (Figure 5d). In addition, *Rnase3a* increased significantly in liver and blood, but decreased in gut at 12 hpi compared to 6 hpi (*p* < 0.01).

## 4. Discussion

RnaseA has been widely studied in the context of biological activities, molecular evolution, structural function and physiological diseases in mammals [43], amphibians [44], primates [45], and teleosts [7]. Many members of the RnaseA superfamily have antibacterial and antiviral activities. It has been reported that human Rnase 2, 3, 5 and 7 [46], chicken Rnase A-2 [47] and mouse Rnase4 [48] possess bactericidal activity. Rnase1-3 and 5 from zebrafish (*D. rerio*) and Rnase1-2 from Atlantic salmon (*S. salar*) have angiogenic and bactericidal activities [3,7]. Our previous study found that Rnase1 of *M. amblycephala* has digestive and antibacterial activity [31]. The present study reports *Rnase2* (six) and *Rnase3* (three) genes in the blunt snout bream genome, and their expression in healthy and bacterially challenged *M. amblycephala*. Only three copies of *Rnase2* and one *Rnase3* are expressed, but they have different patterns of expression. *Rnase3* are more susceptible to *A. hydrophila* infection than *Rnase2.*

In this research, multiple-protein-sequence alignment of *M. amblycephala* and *D. rerio* homologs showed that protein sequences are mostly similar to each other, consistent in size (133–150 amino acids) and possessing the characteristics of the RnaseA superfamily. As opposed to most mammalian Rnases, which have eight conserved cysteines, non-mammalian Rnases, including fishes, have only six [6]. Catalytic residues of Rnase2 and Rnase3 are located in the first and second α-helices and sixth β-strands. The N-terminal domain (1–45) of human Rnase3 includes the main determinants for the protein’s antimicrobial activity [49], so we speculate that *M. amblycephala* Rnase3 antimicrobial activity may be related to the N-terminus, and the helical extension of the N- and C-termini may provide a flexible structure. It has been suggested and experimentally verified in several antibacterial Rnases that positively charged amino acid residues are important for the disruption of negatively charged bacterial cell membranes, and thus are crucial for the bactericidal activity [27]. Indeed, all known bactericidal Rnases, including mammalian Rnase3, 5, 6 and 7, as well as chicken Rnases, have relatively high isoelectric points [28]. As the isoelectric points of *M. amblycephala* of Rnase2 and 3 are relatively high (Figure 1), this suggests that these Rnases may also be bactericidal but requires further assessment. The *Rnase1* gene has multiple copies in mammals, especially in foregut-fermenting monkeys and bovines, suggesting that the duplication of the *Rnase1* gene was an adaptation to their digestive physiology [45,50]. However, there is no genetic duplication of *Rnase1* in teleosts. The two groups (fish and mammals) are divided into two distinct clades in the NJ tree of Rnase2 and Rnase3 which is similar to Rnase1 (Figure 3) [31], In addition, the two genes (*Rnase2* and *Rnase3*) are divided into two distinct clades in the NJ tree of mammals and fishes in our studies (Figure 3), which is consistent with studies reported [2,16]. *Rnase2* of *M. musculus* has two homologous genes, suggesting that the duplication of the *Rnase2* gene was an adaptation to their host defense [51]. *Rnase2* and *3* of other animals don’t have genetic homology. In this study, we found that *M. amblycephala* eosinophils comprise nine genes (*Rnase2a*, *Rnase2b*, *Rnase2c*, *Rnase2d*, *Rnase2e*, *Rnase2f*, *Rnase3a*, *Rnase3b* and *Rnase3c*), but *D. rerio* and *S. salar* possess only one copy of each. There are also many gene duplications of *Rnase1*, *Rnase4*, *Rnase5* and *Rnase6* in Chiroptera [52]. It shows that they have unique evolutionary patterns and functional differentiation. Expression profiles of genes can also be strongly influenced by gene duplications. qPCR was used to further explore the expression patterns of these nine genes.

Our qPCR analysis showed that *Ma-Rnase2* and *3* genes have differential expression patterns. *Rnases2b*, *2c*, *2e* and *3a* were not expressed in the early stages of development, but are weakly expressed at 120 and 140 hpf, which is similar to *D. rerio* [6]. *Rnase2b*, *Rnase2c* and *Rnase2e* were highly expressed in the endocrine tissues (kidney, liver and spleen) and heart of one-year-old fish, and highly expressed in liver, spleen, kidney of two-year-old fish. In addition, *Rnase3a* was most strongly expressed in the heart and testis of one-year-old fish, whereas in two-year-old fish it was highly expressed in all studied tissues, except for the brain and muscle. This expression pattern is different from the one reported for *Rnase2* and *Rnase3* orthologs in adult *D. rerio*: *Rnase3* is highly expressed in heart and gut, but *Rnase2* weakly expressed in heart, liver and gut. However, neither of the two orthologs was expressed in the brain tissue of either of the two species (*D. rerio* and *M. amblycephala*). *Rnase2* of *D. rerio* has a weak digestive activity and antimicrobial activity, whereas *Rnase3* has high antibacterial activity against both gram-positive and gram-negative bacteria. Compared with *D. rerio*, *Rnase3* of *M. amblycephala* has higher expression and stronger antibacterial activity. *Rnase2* in adult *S. salar* is mainly expressed in the thymus, but no expression was detected in other tissues; functionally, Ss-*Rnase2* has strong antibacterial and angiogenic activity [7]. *Rnase2* and *Rnase3* expression patterns in tissues may reflect the variability in their functions in different fish species, as well as in different individual animals. High *Rnase2* and *Rnase3* expression in different tissues might indicate multiple functions. As mentioned in our study, high expression in the thymus indicates that a gene may be involved in immunity-related functions; in the pancreas, it may possess digestion activity as an RNA-degrading enzyme; in the gut, it may be associated with intestinal microbiota [29]. Indeed, the existence of multiple copies of *Rnase2* and *Rnase3* may make their biological activity more powerful and promote the evolution of new protein functions [53]. Only three copies of *Rnase2* and one *Rnase3* are expressed. The specific expression profiles of different *Ma-Rnase2* and *3* paralogs indicate multiple functions, such as RnaseA activity, antibacterial and antiviral activity, etc. Therefore, we conducted a bacterial infection experiment to further investigate the antibacterial activity of *Rnase2* and *Rnase3*.

*Rnase2b* and *Rnase2c* mRNA expression after the *A. hydrophila* challenge was induced in all five *M. amblycephala* tissues at 12 hpi. *Rnase2e* is significantly (*p* < 0.01) up-regulated in kidney at 12 hpi after *A. hydrophila* infection, which could reflect the fact that kidney, as a major immune organ in fish, is more susceptible to *A. hydrophila* infection than liver and spleen. *Rnase3a* was up-regulated in liver, gut and blood 12 hpi after *A. hydrophila* infection. Much higher up-regulated in more tissues observed for *Rnase3* in comparison to *Rnase2* (after *A. hydrophila* infection) could be an indication that *Rnase3* has a stronger antibacterial activity than *Rnase2*. This would be consistent with their corresponding functions in other animals: *Rnase3* has a predominantly antimicrobial activity in zebrafish and humans [45], whereas *Rnase2* has a predominantly antiviral activity in humans [54]. Previous studies have shown that *RNase2* plays an important role in the activation of dendrocytes, immune response modulation, and TLR2 activation [55]. A novel antibacterial activity emerged in *Rnase3* after its duplication [28]. Rnase3 is an antimicrobial protein secreted in response to infection, and is critical for neutralizing bacterial lipopolysaccharides (LPS) and inhibiting tumor necrosis factor production in human macrophages [56]. 

## 5. Conclusions

In conclusion, we have characterized the structure, expression and response to a bacterial challenge of *Ma*-*Rnase2* and *Ma-Rnase3* from the molecular aspect. This study demonstrates preliminarily that, although *Rnase2* and *Rnase3* are very similar, they might remarkably differ in their biological activities. Rnase3 is more susceptible to *A. hydrophila* infection than Rnase2. In following studies, we will focus on the protein expression and biological function of *Ma*-Rnase2 and *Ma*-Rnase3; especially on the antibacterial mechanism of Rnase3 and experimental studies of bacterial infection both *in vitro* and *in vivo*. This will provide a more clear and comprehensive picture of functions and evolutionary history of Rnase2 and Rnase3 in *M. amblycephala*.

## Figures and Tables

**Table 1:** Primers for *Megalobrama amblycephala Rnase2* and *Rnase3* expression analysis.

Primer Name	Primer Sequence (5′-3′)	Fragment Length
*β-actin*	F:ACCCACACCGTGCCCATCTA R:CGGACAATTTCTCTTTCGGCTG	204
*Rnase2a*	F:GTCAACCACCAGACCAAAT R:AAGGCTGAATGCTCCTAAAC	237
*Rnase2b*	F:GATGGCTGCAAACCTGTCA R:CGAGTGGACTTCTTCCCTTT	194
*Rnase2c*	F:GATATGACCGTGCAGAAGTG R:TCTGTATTTACAGTTTGGGTGT	240
*Rnase2d*	F:CCTTCACTCACGGACAACC R:GCACTGACGACCATTTACAT	140
*Rnase2e*	F:AGAGGAGGAACTCGACTGAG R:AGCCTTTATCACAAGCCAAC	157
*Rnase2f*	F:AAGCAATTTGTGGCAGAG R:GGAGTTCCCTTAGTTAGTTTAG	133
*Rnase3a*	F:AGGCAAGCGGATGGAAAG R:CACCATAATGTACTGGGAGACC	166
*Rnase3b*	F:AATTAAAGCCGTTTGTAAGG R:ACCATAATGTACTGGGAGACC	193
*Rnase3c*	F:TTGTCCACTTACAGCCAGAG R:AACCGTTGTTATCTTCTTTGC	256

## Supplementary Materials

The following are available online at http://www.mdpi.com/2073-4425/9/2/95/s1. Figure S1: Nucleotide sequences of *Megalobrama amblycephala Rnase2a*, *Rnase2b*, *Rnase2c*, *Rnase2d*, *Rnase2e*, *Rnase2f*, *Rnase3a*, *Rnase3b* and *Rnase3c*, Figure S2: The 3D protein structure prediction for *M. amblycephala* different Rnases, Table S1: Number of basic amino acids of *Rnase1* to *Rnase3* in fish Rnases, as well as *Homo sapiens* and *Bos taurus* Rnases known to be bactericidal, Table S2: The Ct values of *Rnase2a*, *Rnase2d*, *Rnase2f*, *Rnase3b*, *Rnase3c* and *β-actin* at different stages of development and growth.
