# Supplementary material for: Differential Expression of Six Rnase2 and Three Rnase3 Paralogs Identified in Blunt Snout Bream in Response to Aeromonas hydrophila Infection
Source: Genes (Basel). 2018 Feb 14;9(2):95. doi: 10.3390/genes9020095 (PMC5852591; doi:10.3390/genes9020095)
Supplement: Supplementary file 1 [file genes-09-00095-s001.pdf]

Supplementary Materials:

>*Rnase2a*
ATGGAGATTCATCAGTCTGCTGTGATTCTGCTGCTGATCTTGATTGTTCC
ATCCTTCACTCATGGTCAACCACCAGACCAAATAATGCACAGCTATATAA
AATTCCTCAATCAGCATTTAGGTCCTGATATGACTGAGCAGAAGTGTGAC
AGTGAAATGAGAAAGAGAGGCATCACTGTAAATGATAATGGCTGCAAACC
TGTCAACACCTTCATACAAGCAAATTCAAATCAGATTAAAGCTGTTTGTG
GCAATGGAGGAATTCCACATGGCAATAACTTGTTTAGGAGCATTCAGCCT
TTTCCAGTGATCACATGCACATTAAAATGTGGAGTGAGATATCCAAGATG
TGAATATGGTAAAGGGAAGAAGTCCACTCGTTACATTGTGTTGAAGTGTG
TTGAAGGCTGGCCTGTACATTATGACGAAGGCATAATTAAAGCTGGTGGC
CTATTTAGCTGGCTTTAA
>*Rnase2b*
ATGGAGATTCATCAGTCTGCTGTGATTCTGCTGCTGATCTTGAGTGTTTC
ATCCTTCACTCATGGACAACCAGACAATATAAAGCCCCGCTATATAAAAT
TCCTCAATCAGCATTTCGGTCCTGATATGACTGAGCAGAAGTGTACCAGT
GAAATCAGACAGAGAGATATCACTGGAAATGATGATGGCTGCAAACCTGT
CAACACCTTCATACAAGCGAATTCAAATGATATCAGAGCAGTTTGTGGCA
CAGGAGGAACTCCACAGGGAGGGGACCTGTTTAAGAGCAACCAGCCTTTT
CCGGTGATCACATGCAATTTACAAAGTGGGCAGAGACCCCCAAGATGTGA
ATATGGTAAAGGGAAGAAGTCCACTCGTTACATTGTGTTGGGGTGTGCAG
AAGGCTGGCCTGTACATTATGAAGAAGGCATACCTGATACAAAGAAGACA
GGCTGA
>*Rnase2c*
ATGGAGATTCATCAGTCTGCTGTGATTCTGCTGCTGATCTTGAGTGTTTC
ATCCTTCACTCACGGACAACCACCAGCACATATAAAGCCCCGCTATGAAA
AATTCCTCAAACAGCATTACAATCCTGATATGACCGTGCAGAAGTGTGAC
ACTGTAATTCGTGGAAATAGAGATTTTATTCACAGTGAAACTGAAAATGG
CTGCAAAAAAATCAACACCTTCATAAAAGCAAATTCAAATGATATTAAAG
CAGTTTGTGGAAAAGCAGGAAAACCGTACCGCAATATGGTTGCGAGCACC
AAGCCTTTTTCTGTGGTCACCTGCAAATTACACAGCGGGGATACACACCC
AAACTGTAAATACAGAGGGGTCCAGGACACTCGTTATATAGTGTTGGCGT
GTGAACATGGCTGGCCTGTACATTATGATGAAGGCATATATAAGCCTAAT
GGAAGCTAA
>*Rnase2d*
ATGGAGATTCATCAGTCTGCTGTGATTCTGCTGCTGATCTTGAGTGTGTC
ATCCTTCACTCACGGACAACCAGCAGATATATGGCCACGCTATGAAAAAT
TCCGCAATCAGCATGTGATTCCTGATATGACTGAAGATATGTGTACCAGA
GTAATCAAAAGCAGACGCATCAATGTAAATGGTCGTCAGTGCAAACCTGT
CAACTCCTTCATAGCAGCAAATGAACATCAAATTAATGAAGTTTGTGGCA
GAGGAGGAACTCCACAGGGTGGGGACCTGTTTGAGAGCAACCAACGATTT
TTTGTAGTCAAGTGTACATCAAGTGGTGGGATGTATTATCCAAACTGTCA
ATATTCTGGGAGGAGTTACACTAGTAAGATTGTGTTGCAGTGTGATAGAG

GCTGGCCTGTACATTATGTAAGGTAA
>*Rnase2e*
ATGGAGATTCATCAGTCTGCTGTGATTCTGCTGCTGATCTTGAGTGTTTC
ATCCTTCACTCACGGACAACCAGCAGATATATGGCAACGCTATGTAAAAT
TCCTCAATCAGCATGTGGATCCATTTATGACTGTGAATGATTGTACCAGT
AGAATCGAAAACAGAGACATCACTGGAACTAATGGTGCCTGCAAACCTGT
CAACTCCTTCATAGTAGCAAATGCAGATACAATTAAAACAGTTTGTGGCA
GAGGAGGAACTCGACTGAGGAATGATCGAAACCTGTTTGAGAGCGACCTA
CGATTTTTTGTGGTCAAGTGTAATATAAAAACAATTAAGAATGGAAAGTG
TGAATATGTAGGGAAGGGTTACACTAGAAAGATTGTGTTGGCTTGTGATA
AAGGCTGGCCTGTACATTATGAAAGGTAA
>*Rnase2f*
ATGGAGATTCATCAGTCTGCTGTGATTCTGCTGCTGATCTTGAGTGTTTC
ATCCTTCACTCACGGACAACCAGCAGATGTAAGGCGACGCTATGAAAAAT
TCCTCAATCAGCATGTGGATCCATTTATGACTGTGAATGATTGTACCAGT
AAAATCGAAAACAGAGATATCACTGCAACTGATGGTGACTGCAAACCTGT
CAACTCCTTCATAGTAGCAAATGAAGATACAATTAAAGCAATTTGTGGCA
GAGGAGGAACTCGACTGAGGAATGATCAAAACTTGTTTGAGAGCAACCTA
CCATTTTTTGTGGTCAAGTGTACAATAAAAACTAGTAAGTTTACTACTAA
ACTAACTAAGGGAACTCCAAAGTGTGAATATGTAGGGAAGGGTTACACTA
GTAAGATTGTGTTGGCTTGTGATAGAGGCTGGCCTGTACATTATGAAAGG
TAA
>*Rnase3a*
ATGAAGATCCATCAGTCTACTGTGATTCTGCTGCTGGTTATATGTGCACC
ATTGTCCACTTACAGCCAGAGTTATGGGGCTTTCAAAAACAAACATGTGG
GTCCACTCAACATGGCTGGACGGGATTGTACCGCTGTGATCGAAAATAGA
CGCATCAATAAAAAAAACTGTAAATGCAAAAAAATAAACACCTTCATAAT
GGCAACTGAACAGCAAATTAAAGATGTTTGTAAGACAGGAAAAAGGCAAG
CGGATGGAAAGACCTTCATAAGCAATACTGTTTTTGATGTGATTGATTGT
ACTCTTAAAACAGAAGATAACAACGGTTGTAAATATAAGGATGATGTTAA
ACCAAAAACAAACACAATTACTTTGACATGTGAAAATGGTCTCCCAGTAC
ATTATGGTGAAGCAAAATAG
>*Rnase3b*
ATGAAGATCCATCAGTCTACCGTGATTCTGCTGCTGGTTATATGTGCACC
ATTGTCCACTTACAGCCAGAGTTATGAGGCTTTCATAAACAAACATGTGG
GTCCAAACAAGATGACTGGACGGGATTGTACCGCTGTGATCGAAAATAGA
CTCATCAATGAAAACCGCGAATGCAAACAAATAAACACCTTCATAATGGC
AACTGAAGATGAAATTAAAGCCGTTTGTAAGGATGGAAAAAAGCAAGCGG
ATGGAAAGACCTTCATAAGCAATACTGAATTTGATGTGATTGATTGTACT
CTTAAAAGAAAAGATAACAACGGTTGTAAATATAAGGATGATGTTAAACC
AAAAAAAACCACAATTACTTTGACATGTGAAAATGGTCTCCCAGTACATT
ATGGTGAAGCAAAATAG
>*Rnase3c*
ATGAAGATCCATCAGTCTACTGTGATTCTGCTGCTGGTTATATGTGCACC

ATTGTCCACTTACAGCCAGAGTTATGAGTTTCAAAAAAAACATCTGGCTC
CAAACGGGATCACTGACAATGATTGTACCACCGAGATCAAAAATAGACAA
ATCAATAAGTGCAGAAAAATACACACCTTCATAAAAGCAAATGAAAATGA
AATTATACATGTTTGTGATAATGAAAAAAAGCATCAGAATTTGCCCTATA
TGATAAGTGCTACATTTAATGTGATTGATTGTGCTGGCAAAGAAGATAAC
AACGGTTGTAATTATACTCATGTTGAACCAAATAGAAAAACATTTATATT
GACATGTGAAAAAGGTTTCCCAGTACATTATGGCGAATCCACCTTAAAGT
AG

Figure S1. Nucleotide sequences of *Megalobrama amblycephala Rnase2a*, *Rnase2b*, *Rnase2c*, *Rnase2d*, *Rnase2e*, *Rnase2f*, *Rnase3a*, *Rnase3b* and *Rnase3c*.

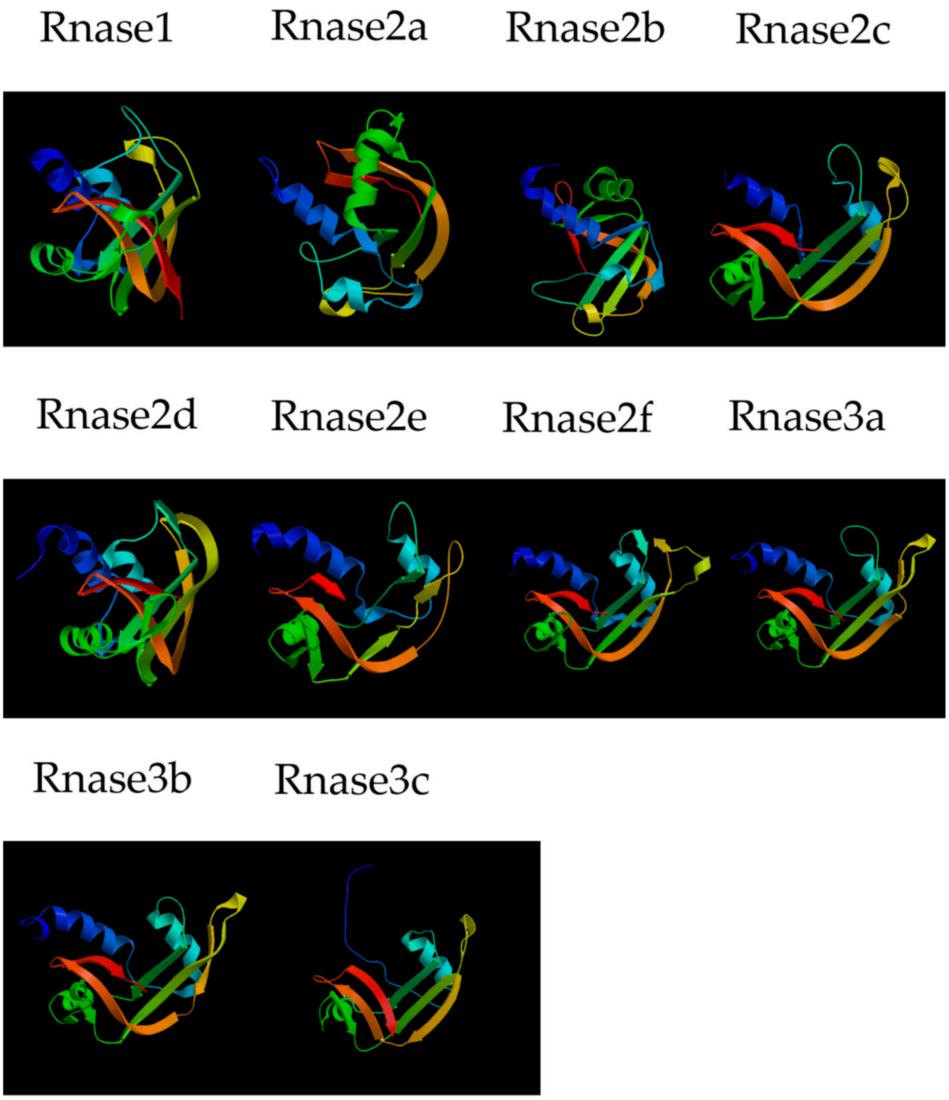

Figure S2. The 3D protein structure prediction for *M. amblycephala* Rnase1, Rnase2a, Rnase2b, Rnase2c, Rnase2d, Rnase2e, Rnase2f, Rnase3a, Rnase3b and Rnase3c modeled using the SWISS-MODEL automatic protein modeling server ProMod3 program. The light blue, blue and green ribbons represent the three α-helices and ribbons with other colors representing the six β-strands.

Table S1. Number of basic amino acids of Rnase1 to Rnase3 in fish Rnases, as well as *Homo sapiens* and *Bos taurus* Rnases known to be bactericidal.

Protein	Number of lysines	Number of arginines	Total
Ma-Rnase1	3	17	20
Ma-Rnase2a	11	6	17
Ma-Rnase2b	10	7	17
Ma-Rnase2c	14	6	20
Ma-Rnase2d	5	11	16
Ma-Rnase2e	10	10	20
Ma-Rnase2f	12	9	21
Ma-Rnase3a	17	4	21
Ma-Rnase3b	16	4	20
Ma-Rnase3c	12	3	15
Dr-Rnase1	4	15	19
Dr-Rnase2	12	10	22
Dr-Rnase3	15	5	20
Ol-Rnase1	7	11	18
Ol-Rnase2	9	7	16
Ol-Rnase3	14	7	21
Om-Rnase1	11	8	19
Om-Rnase2	11	5	16
Ss-Rnase1	7	10	17
Ss-Rnase2	13	2	15
Hs-Rnase1	8	10	18
Hs-Rnase2	4	8	12
Hs-Rnase3	1	19	20
Bt-Rnase1	10	4	14
Bt-Rnase2	3	12	15
Bt-Rnase3	3	12	15

Ma: *Megalobrama amblycephala*, *Dr*: *Danio rerio*, *Ol*: *Oryzias latipes*, *Om*: *Oncorhynchus mykiss*, *Ss*: *Salmo salar*, *Hs*: *Homo sapiens*, *Bt*: *Bos taurus*.

Table S2. The Cycle threshold (Ct) values of *Rnase2a, Rnase2d, Rnase2f, Rnase3b, Rnase3c* and *β-actin* at different stages of development and growth. There are three replicates per each sample: hpf = hours post fertilization.

early developmental stages	*β-actin*	*Rnase2a*	*Rnase2d*	*Rnase2f*	*Rnase3b*	*Rnase3c*
0hpf 1	24.83	36.6	34.65	31.82	35.59	31.55
0hpf 2	25.87	36.92	34.31	32.61	35.47	31.71
0hpf 3	25.39	36.3	34.5	31.91	35.73	30.96
16hpf 1	24.37	32.79	32.83	32.09	37.53	33.64
16hpf 2	23.77	33.1	33.2	31.6	37.55	33.65
16hpf 3	24.4	33.52	34.53	31.23	37.64	33.66
38hpf 1	21.89	33.8	32.35	31.87	36.53	33.5
38hpf 2	22.33	33.93	32.55	32.59	37.98	32.69
38hpf 3	21.48	33.83	32.25	30.71	36.64	34.25
48hpf1	17.69	33.43	30.71	30.39	32.57	33.3
48hpf 2	17.74	33.63	30.25	30.74	33.75	32.36
48hpf 3	18.35	33.95	30.54	30.88	33.96	34.3
88hpf 1	20.73	31.67	32.43	31.31	35.4	33.65
88hpf 2	20.83	31.68	31.85	32.69	34.5	33.63
88hpf 3	20.85	31.69	32.14	31.98	35.6	33.7
120hpf 1	17.23	32.86	30.25	30.39	35.44	34.61
120hpf 2	17.4	32.64	30.46	30.45	35.65	33.57
120hpf 3	17.81	32.42	30.33	30.37	35.55	33.81
140hpf 1	19.22	32.2	30.57	31.31	34.88	33.85
140hpf 2	18.84	33.27	30.02	31.27	34.78	34.56
140hpf 3	18.68	34.48	30.57	31.32	35.6	33.23

one-year-old tissues	*β-actin*	*Rnase2a*	*Rnase2d*	*Rnase2f*	*Rnase3b*	*Rnase3c*
heart 1	18.20	31.56	30.97	30.67	33.82	32.67
heart 2	17.79	31.87	31.73	30.98	33.8	32.75
heart 3	17.94	31.75	30.86	30.74	33.18	32.29
liver 1	19.42	30.54	30.65	36.02	34.18	34.52
liver 2	18.64	30.51	30.35	36	33.77	34.61
liver 3	18.41	30.20	31.34	36.17	34.81	34.95
spleen 1	14.95	30.58	30.89	32.33	30.99	31.5
spleen 2	15.43	30.40	31.68	32.76	30.72	31.42
spleen 3	14.89	30.15	31.87	32.9	30.75	31.62
kidney 1	15.29	33.40	30.82	30.77	32.55	30.65

kidney 2	15.52	33.69	30.60	30.64	32.79	30.83
kidney 3	15.54	33.74	30.28	30.88	32.97	30.41
brain 1	16.55	30.32	30.60	33.23	35.91	32.26
brain 2	16.47	30.53	31.37	33.75	34.91	31.95
brain 3	16.60	30.49	31.61	33.72	34.55	31.67
gut 1	18.61	30.23	30.54	32.91	34.06	30.87
gut 2	19.67	30.35	31.25	32	34.74	30.96
gut 3	18.61	30.91	30.30	32.68	34.99	30.67
muscle 1	18.93	30.34	32.61	33.49	32.35	33.59
muscle 2	18.98	30.29	31.77	33.19	31.85	33.92
muscle 3	17.56	29.91	31.58	32.83	31.67	33.42
testis 1	20.41	30.40	30.07	31.91	30.21	31.22
testis 2	20.24	30.67	30.52	31.85	30.76	31.97
testis 3	20.69	30.38	30.57	31.4	30.47	31.53

two-year-old tissues	*β-actin*	*Rnase2a*	*Rnase2d*	*Rnase2f*	*Rnase3b*	*Rnase3c*
heart 1	18.64	35.54	30.99	36.27	34.65	31.99
heart 2	19.97	35.23	30.31	37.94	36.75	32.66
heart 3	19.32	35.6	30.48	37.56	35.74	32.39
liver 1	19.94	34.95	33.43	30.62	36.71	31.67
liver 2	18.83	34.8	33.35	29.52	36.63	31.43
liver 3	19.26	34.62	33.25	30.46	36.71	31.52
spleen 1	17.74	34.44	33.8	31.07	34.99	31.23
spleen 2	17.56	34.21	34.47	30.90	34.93	31.9
spleen 3	17.66	34.53	34.38	30.55	34.36	31.81
kidney 1	17.95	33.76	31.93	34.66	33.55	30.62
kidney 2	18.34	33.82	31.34	33.38	33.21	30.98
kidney 3	16.98	33.52	31.42	34.68	33.82	30.82
brain 1	17.30	30.47	32.28	34.74	35.59	31.71
brain 2	17.53	30.48	32.22	34.76	35.65	31.88
brain 3	16.98	30.94	32.59	34.5	35.47	31.68
gut 1	17.63	30.92	32.8	31.26	36.42	32.62
gut 2	18.41	30.83	32.7	31.64	36.95	32.3
gut 3	18.42	30.39	32.61	32.99	36.64	32.88
muscle 1	18.35	33.47	32.84	30.62	35.77	33.47
muscle 2	18.25	33.65	32.48	30.24	35.43	33.35
muscle 3	17.68	33.17	32.48	31.4	34.8	33.19

testis 1	17.55	33.95	30.15	30.38	30.53	31.44
testis 2	18.63	34.24	30.82	31.57	30.7	31.54
testis 3	18.82	34.66	30.82	30.56	30.95	31.64
